# Immunoevasion strategies for African swine fever virus: Modulation of antigen presentation pathways

**DOI:** 10.1080/21505594.2025.2541711

**Published:** 2025-08-15

**Authors:** Penghao Lv, Zhichao Wang, Haowei Chen, Yanru Chen, Hanlin Liao, Kaiyue Wei, Shuying Lv, Min Cui

**Affiliations:** a State Key Laboratory of Agricultural Microbiology, College of Veterinary Medicine, Huazhong Agricultural University, Wuhan, Hubei, China; b Cooperative Innovation Center for Sustainable Pig Production, Hubei Jiangxia Laboratory, Huazhong Agricultural University, Wuhan, Hubei, China; c Department of English Teaching and Research Section, Dongying Vocational College, Dongying, China

**Keywords:** African swine fever, African swine fever virus, antigen presentation, adaptive immunity, vaccines

## Abstract

African swine fever virus (ASFV), a highly lethal pathogen, poses a catastrophic threat to global pork production. While some commercial vaccines exist, their availability remains regional limitations. This review investigates the mechanistic interplay between ASFV and antigen-presenting cells (APCs), with a particular focus on viral evasion strategies that disrupt antigen presentation pathways. Although current understanding of ASFV immunology has largely focused on monocyte/macrophage infection, we provide a systematic analysis of ASFV-mediated immunosuppression across various APCs, including dendritic cells (DCs), B lymphocytes, and γδ T cells, and compare these mechanisms with those employed by other viral pathogens. Key objectives include: (1) synthesizing ASFV-induced dysregulation of antigen presentation, (2) identifying critical gaps in APC-virus interactions, and (3) guiding vaccine development priorities. By highlighting understudied DC-ASFV interplay and potential molecular targets, this work provides a strategic framework for next-generation vaccine design based on host-pathogen conflict at the antigen presentation interface.

## Introduction

ASF is a highly contagious viral disease that severely impacts swine populations; First identified in Kenya in 1921, the disease has since spread to Europe, South America, and Southeast Asia [1–6]. ASF is pathologically characterized by multisystemic haemorrhagic necrosis, prominently affecting lymphoid tissues, the renal parenchyma, and the cardiac architecture. The key pathological manifestations include splenomegaly and haemorrhagic diathesis [6,7]. With a mortality rate that can reach 100%, ASF has devastating effects on domestic pig (Sus scrofa) populations and the pork industry worldwide, representing one of the major threats to the global farming industry.

ASFV belongs to the genus *Asfivirus* in the family *Asfarviridae* [8]. The ASFV genome ranges from 170 to 193 kbp in size and contains 150 to 190 open reading frames (ORFs), encoding more than 180 proteins. However, the functionality of most of the proteins remains unclear [9]. ASFV is classified into 24 genotypes based on the variations in the terminal nucleotides of the *B646L* gene, which encodes the capsid protein p72 [10]. In Africa, genotypes I and II are the primary strains that have spread to other regions although 24 genotypes exist [11]. Genotype II (including the Georgia 2007/1 strain and its derivative variants) remains the most prevalent strain in Europe [12]. The current prevalent strains in China are mainly genotype II [13], and there are also some recombinant strains of genotype I and type I/II [14,15]. ASFV isolates are typically classified as highly virulent, moderately virulent, or low virulent, with clinical presentations ranging from acute haemorrhagic fever and death to mild clinical signs and chronic infection [16,17].

Over the past few decades, various vaccination strategies have been explored, including inactivated vaccines, DNA vaccines, subunit vaccines, adenovirus-vectored vaccines and live attenuated vaccines (LAVs). While most current vaccine approaches fail to induce effective protective immunity against ASFV, LAVs demonstrate efficacy but raise significant safety concerns that require careful evaluation [18–20].

ASFV infection activates both innate and adaptive immune responses, with the adaptive arm being pivotal in mediating protection against viral pathogens [21]. The humoral immune system generates virus-specific antibodies through B cell activation and differentiation into antibody-secreting plasma cells, while CD8^+^ cytotoxic T lymphocytes (CTLs) eliminate infected cells through MHC-I-restricted cytotoxic mechanisms [22]. However, ASFV has evolved sophisticated immune evasion strategies that subvert both innate and adaptive immunity, posing significant challenges for vaccine development [23,24].

This review delineates the interplay between ASFV and APCs while hypothesizing the putative mechanisms underlying ASFV-mediated inhibition of antigen presentation processes, thereby establishing a conceptual framework to inform next-generation vaccine development strategies.

## Antigen presentation in viral infection

Adaptive immunity serves as a critical defence mechanism against pathogenic infections, playing a pivotal role in immune protection [25]. At the core of adaptive immunity lies antigen presentation, which serves as the fundamental basis for initiating immune responses. Notably, ASFV employs sophisticated immune evasion strategies by inhibiting the antigen presentation process, thereby effectively suppressing the activation of adaptive immunity [26].

Antigen presentation occurs through three primary pathways: the endogenous (MHC-I) pathway, the exogenous (MHC-II) pathway, and the cross-antigen presentation pathway. MHC-I molecules are expressed on all nucleated cells and present endogenous antigens to CD8^+^ T cells, whereas MHC-II molecules are exclusively expressed by professional antigen-presenting cells (APC), including dendritic cells, macrophages, and B cells, which present exogenous antigens to CD4^+^ T cells [27]. Among these, DCs, macrophages, and B cells serve as the principal APCs that play indispensable roles in the innate and adaptive immune response for the clearance of viruses such as ASFV [28–32]. Given the central immunological role of APCs, comprehensive characterization of ASFV-mediated modulation of these cells represents a crucial step towards developing effective vaccine strategies against this pathogen.

### Central role of macrophages in ASFV infection

Monocytes and macrophages represent a functionally diverse cell population that orchestrates critical innate and adaptive immune responses [33]. During innate immune responses, macrophages detect pathogens, initiate inflammatory cascades, and recruit effector cells to infection sites. In adaptive immune responses, they contribute to antigen presentation and production of immunomodulatory cytokines that activate lymphocyte responses, thereby bridging innate and adaptive responses [34]. Monocytes and macrophages are the primary target cells for ASFV infection, supporting the complete viral replication cycle, including the genome replication, virion assembly, and subsequent release of progeny virions [35]. Consequently, the interaction of ASFV with macrophages is fundamentally determinative of viral immunopathogenesis and clinical disease outcomes.

Transcriptomic analysis of porcine alveolar macrophages (PAMs) following infection with genotype II ASFV-HLJ/18 at an MOI of 1 revealed significant changes in gene expression. Among the 536 upregulated and 838 downregulated differentially expressed genes (DEGs), the majority were consistently altered across multiple time points postinfection [36]. Another study demonstrated that infection with ASFV-HLJ/18 at an MOI of 1 led to the sustained upregulation of 114 genes and the downregulation of 233 genes in PAMs from 6 to 24 hpi [37]. The key pathways affected by ASFV infection include the interferon (IFN) response, inflammatory signalling, apoptosis and other immune regulatory pathways.

#### Inhibition of the interferon response and signaling by ASFV

Although the IFN-mediated antiviral response plays an indispensable role in viral clearance [38], ASFV has evolved multiple strategies to antagonize the host IFN-mediated immune response, enabling its replication and spread within the host. Indeed, ASFV encodes numerous multifunctional proteins that manipulate and evade the host antiviral response by specifically targeting components of the IFN signalling pathway.

For example, the ASFV multigene family (MGF) 360-9L interacts with signal transducer and activator of transcription (STAT) 1 and STAT2, and degrades STAT1 and STAT2 via the apoptosis and the ubiquitin – proteasome pathway. Consequently, the activation of IFN-β signalling was inhibited. Notably, a recombinant ASFV strain with the MGF360-9L gene deletion was exhibited significantly reduced virulence compared with the parental strain [39]. Moreover, MGF505-7R, a member of the MGF505 family, strongly inhibits IFN-β promoter activation, and further study revealed that MGF505-7R interacts with the nuclear translocation of interferon regulatory factor 3 (IRF3), blocking its nuclear translocation and thereby inhibiting type I IFN production [40]. Additionally, MGF505-7R has been reported to interact with IRF9, suppressing the formation of the interferon-stimulated gene factor 3 (ISGF3) trimer and inhibiting its nuclear translocation, further dampening IFN-β production [41]. These findings demonstrate that a single ASFV protein can employ multiple mechanisms to inhibit IFN signalling. Table 1 summarizes recent research advances in understanding the molecular mechanisms by which ASFV-encoded proteins regulate IFN responses.

**Table 1.** Characterized ASFV-encoded immunomodulatory proteins that target interferon (IFN) pathways.

Viral gene	Molecular mechanisms	Impact on virulence	Reference
K205R	Interacting with the IFNAR1 and IFNAR2.	ND	[42]
MGF360-4L	Interacting with MDA5; Suppressing the phosphorylation of IRF3.	Attenuated	[43,44]
DP71L	Interacting with the STING.	ND	[45]
MGF505-6R	Impairing the phosphorylation and nuclear translocation of IRF3.	ND	[46]
I7L	Interacting with STAT1.	Attenuated	[47]
MGF505-2R	Interacting with STING.	Attenuated	[48]
P17	Interacting with STING and IRF3; Binding to TOMM70.	ND	[49–51]
MGF505-4R	Interacting with TRAF3.	ND	[52]
A151R	Interacting with TRAF6.	ND	[53]
B318L	Interacting with STING.	Attenuated	[54]
DP96R	Interacting with IRF3.	ND	[55]
H240R	Interacting with IFNAR1 and IFNAR2. pH240R inhibited the phosphorylation of IRF3 and TBK1	Attenuated	[56,57]
MGF360-10L	MGF360-1L targets JAK1 and mediates its degradation.	Attenuated	[58]
S273R	Disrupting the association of TBK1 and IRF3; Targeting IKKε.	ND	[59–61]
B175L	Interacting with both cGAMP and STING.	ND	[62]
D129L	Interfering with the interaction of p300 and IRF3.	ND	[63]
F778R	Weakening the nuclear accumulation of activated STAT1.	ND	[64]
MGF110-9L	Degrading TBK1.	Attenuated	[65]
A104R	Attenuating STAT1 phosphorylation.	ND	[66]
CD2v	Interacting with STING.	Attenuated	[67]
MGF360-13L	Suppressing the cGAS-STING-IFN-I axis by degrading STING.	ND	[68]
MGF505-7R	Interacting with IRF9 and IRF3.	Attenuated	[40, 41]
L83L	Interacting with cGAS and STING.	ND	[69]
I215L	Inhibiting ISRE activity; Triggering IRF9 degradation.	ND	[70, 71]
E184L	Interacting with STING.	ND	[72]
A137R	Interacting with TBK1.	Attenuated	[73]
M1249L	Interacting with IRF3.	ND	[74]
MGF360-9L	Targeting degradation of STAT1 and STAT2.	Attenuated	[39]
MGF360-14L	Downregulating expression of the IRF3; Interacting with IRF3.	ND	[75]
A528R	Targeting p65 activation and nuclear translocation.	ND	[76]
MGF360-11L	Interacting with TBK1 and IRF7.	ND	[77]
MGF505-11R	Interacting with STING.	ND	[78]
E120R	Interacting with IRF3.	ND	[79]
I329L	Inhibiting TLR signaling.	No impact	[80, 81]
MGF360-12L	Suppressing the nuclear localization of p50 and p65. Interacting with KPNA2, KPNA3, and KPNA4.	ND	[82]
DP148R	ND	Attenuated	[83]
E301R	Interacting with IRF3.	ND	[84]

ND means not done.

#### ASFV modulates NF-κB-mediated inflammatory responses promoting immune evasion and pathogenesis

Inflammation is an important immune defence mechanism that is beneficial for the host to resist infection [6]. However, an imbalance in the inflammatory response can also lead to tissue and organ damage, promoting the occurrence of diseases. ASFV infection induces robust release of proinflammatory cytokines, including interleukin-1 (IL-1), interleukin-6 (IL-6), and tumour necrosis factor-alpha (TNF-α), culminating in a severe cytokine storm [85–88]. This dysregulated immune response is considered one of the primary factors in host mortality. However, differences exist in the regulation of proinflammatory responses between low- and high-virulence strains. While low-virulence ASFV isolates often trigger higher cytokine levels in vitro, highly virulent strains appear to suppress excessive inflammation, potentially evading host immune defences [89].

Recently, several ASFV proteins involved in regulating the activation of NF-κB have been identified. The ASFV protein I10L acts as an inhibitor of the TNF-α- and IL-1β-triggered NF-κB signalling pathway. Specifically, pI10L inhibits IKKβ phosphorylation by reducing the K63-linked ubiquitination of NEMO, a critical step for the activation of IKKβ. Moreover, pI10L interacts with the kinase domain of IKKβ through its N-terminus and consequently blocks the association of IKKβ with its substrates IκBα and p65, leading to reduced phosphorylation [90]. The F317L protein also interacts with IKKβ, suppressing its phosphorylation. This interaction stabilizes IκBα and blocks the activation and nuclear translocation of NF-κB, resulting in decreased expression of various proinflammatory cytokines and increased viral replication efficiency [91]. The MGF505-7R protein inhibits NF-κB activation by binding to IKKα, leading to reduced IL-1β production. ASFVs lacking the MGF505-7R gene exhibit reduced virulence in piglets [40]. Similarly, deletion of the H240R gene decreases infectious viral progeny production due to aberrant virion morphogenesis and increased inflammatory cytokine expression both in vitro and in vivo [92]. The MGF360-12L protein significantly inhibits host mRNA transcription and the promoter activity of IFN-β and NF-κB, as well as the nuclear localization of p50 and p65, by competitively inhibiting the interaction between NF-κB and nuclear transport proteins [82]. The L83L gene expressed early specifically associates with IL-1β; however, deletion of the L83L gene only slightly affects the virulence of ASFV. Further in-depth studies are needed to elucidate how ASFV regulates inflammatory responses, which could provide critical insights into this devastating disease.

#### ASFV has dual roles in regulating apoptosis

Upon viral infection, host cells initiate apoptotic pathways to restrict viral replication and prevent systemic dissemination of the infection. Unfortunately, many viruses have evolved sophisticated countermeasures, encoding antiapoptotic proteins to suppress these host cell death pathways.

CD2v, the principal envelope glycoprotein of ASFV, facilitates STAT3 transcription, phosphorylation, and subsequent translocation into the nucleus. Further research has shown that CD2v interacts with CSF2RA to activate the JAK2-STAT3 pathway and inhibit apoptosis, thereby suppressing apoptosis and ensuring the survival of infected cells, which promotes viral replication [93]. The ASFV-encoded A179L protein demonstrates potent antiapoptotic activity when expressed exogenously in cells [94]. Structural characterization reveals it contains three conserved BCL-2 homology domains (BH4, BH1, and BH2) and localizes to the mitochondria or endoplasmic reticulum [95]. A179L binds to proapoptotic BCL-2 proteins (such as Bid, Bim, and Bad) through their BH3 domains with different affinities, effectively neutralizing their pro-apoptosis activity. This molecular interference blocks cell apoptosis pathways, thereby creating a favourable intracellular environment for prolonged viral replication [96]. Consistently, macrophages infected with A179-deleted ASFV strain exhibit significantly accelerated apoptosis compared to parental strain [97]. The ASFV A224L protein is an analog of the inhibitor of apoptosis (IAP) protein family [98]. Infection with A224L-deleted ASFV strains resulted in significantly enhanced caspase-3 activity and accelerated apoptotic progression. Further studies indicated that the overexpression of the A224L protein could activate the NF-κB pathway, suggesting that this viral protein may dampen apoptosis through the activation of several antiapoptotic genes, such as IAP and BCL-2 family proteins [99].

#### Macrophages in ASFV infection: a double-edged sword of antiviral defense and immunopathological collapse

Monocyte – macrophage lineage cells, ubiquitously distributed across tissues, are essential regulators of tissue morphogenesis, homoeostatic maintenance, remodelling, and repair processes following injury. As the primary cellular targets of ASFV, these cells mediate initial antiviral responses and orchestrate intercellular immune communication [100]. The infection-induced hyperinflammation may drive lymphocytopenia through mechanisms mirroring COVID-19-associated cytokine storms, where excessive TNF-α and IL-6 production triggers self-perpetuating inflammatory cascades leading to lymphocyte depletion and T-cell exhaustion [101]. Similarly, in ASFV infection, the lymphocytopenia likely results from an inflammatory storm where infected macrophages produce excessive proinflammatory mediators that induce lymphocyte apoptosis, thereby paralysing both humoral and cellular immune immunity [85, 102]. Concomitantly, excessive inflammation provokes tissue damage, and releases self-antigens that may breach immune tolerance and trigger autoimmune responses. This dual mechanism combines direct viral cytopathic effects with immune-mediated collateral damage, highlighting the precarious equilibrium between antiviral defence and immunopathology [103]. Notably, the capacity of ASFV to hijack monocyte‒macrophage functions not only enables viral persistence but also destabilizes immune homoeostasis, creating a permissive microenvironment for both viral dissemination and secondary autoimmune complications.

**Figure 1.**
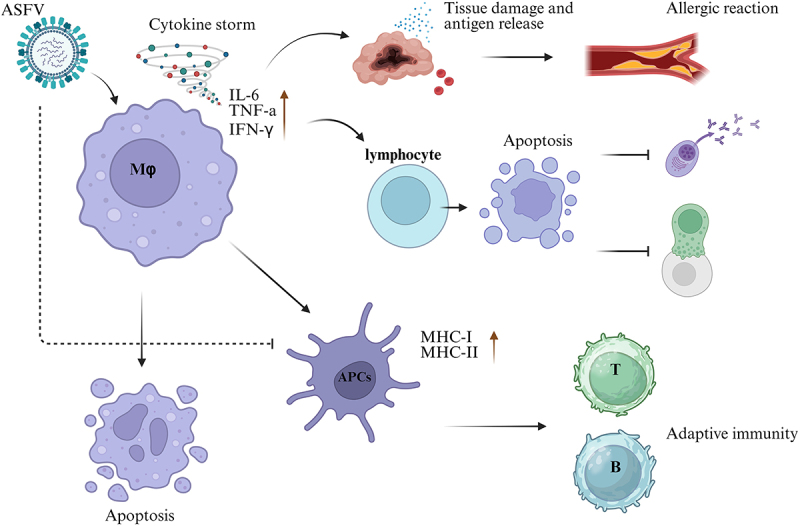
Impact of ASFV infection on Mφ immune responses.

Importantly, experimental evidence has demonstrated that ASFV infection downregulates MHC-II and CD80 costimulatory molecule expression on porcine peripheral blood monocytes, indicating compromised antigen-presenting capacity [104, 105]. Notably, both ASFV-infected and uninfected splenic monocytes exhibit reduced MHC-II expression, suggesting that infection-altered cytokine milieus may mediate bystander impairment of antigen presentation [106]. These findings collectively indicate that ASFV employs dual mechanisms to subvert antiviral immunity: direct targeting of antigen presentation machinery in monocyte‒macrophage, and indirect disruption of lymphocyte survival/activation through cytokine-mediated apoptosis. This multifaceted immunosuppression ultimately delays viral clearance by impeding critical T-cell-dependent immune responses. Figure 1 summarizes the ASFV-mediated immunomodulation in Mφs and its systemic impact on host immune responses.

Notably, a marked difference exists between in vivo and in vitro ASFV infection models: while the former consistently induces severe cytokine storms, the latter often fails to reliably reproduce this phenomenon.

Current in vitro models predominantly utilize macrophage monocultures, which fail to adequately capture the complex multicellular microenvironment of host tissues. In vivo, cytokine storms emerge through intricate intercellular communication networks among immune cells (particularly lymphocytes and dendritic cells) and stromal components (including epithelial and endothelial cells), which are interactions absent in simplified in vitro systems [107, 108]. This implies that the cytokine storm may reflect a secondary immune cascade rather than a direct viral effect. To address this translational gap, future studies should employ physiologically relevant co-culture systems that better replicate critical cellular interactions observed in vivo. In addition, individual differences between different viral isolates and host pigs may also lead to inconsistent results in vitro and in vivo.

ASFV exhibits marked tropism for the cells of monocyte – macrophage lineage, potently increasing the production of proinflammatory factors and triggering a systemic cytokine storm. This inflammatory cascade induces both macrophage apoptosis and depletion of T and B lymphocytes, thereby simultaneously compromising cellular and humoral immunity while causing tissue damage through teh excessive inflammatory cytokines. Concurrently, the release of autoantigens from damaged tissue exacerbates hypersensitivity reactions. Furthermore, ASFV suppresses host adaptive immune responses by downregulating both MHC class I and II molecules, thereby evading immune recognition.

#### Interaction of ASFV with the polarization phenotypes of macrophage subsets

Macrophages can undergo differential activation, polarizing into distinct functional subsets known as M1 and M2 macrophages. M1 macrophages mediate host defence against intracellular pathogens, whereas M2 macrophages primarily contribute to immunosuppression and tissue repair [109]. One study demonstrated that the ASFV Pig/HLJ/2018 strain infected PAMs at an MOI of 1, with transcriptional and protein levels of polarization markers measured from 0 to 48 hpi. The results indicated that after ASFV infection, the transcription levels of M1 cytokines (IL-1β, TNF-α, and IL-6) in PAMs significantly increased, whereas those of M2 cytokines (IL-10 and Arg-1) initially increase but then decreased. These findings suggest that ASFV infection induces the polarization of PAMs towards both the M1 and M2 phenotypes. M1 polarization persisted with prolonged infection time, while M2 polarization exhibited an initial increase followed by decline [110]. Another study revealed that ASFV reduces M1 activation by downregulating the expression of M1-activating receptors, signalling mediators, and transcription factors, while increasing the expression of cytokine antagonists (IL1RN and IL18BP) and the cytokine IL-13 [17].

The differentiation of monocytes into macrophages increased susceptibility to ASFV infection, which is consistent with the finding of previous publications [111]. Compared with the avirulent strain BA71V, the virulent ASFV 22,653/14 strain demonstrated greater infectivity in moM1 macrophages. While IFN-γ or LPS pre-treatment confers resistance against BA71V infection, these treatments have no protective effect against 22,653/14 infection [109]. Importantly, considering the substantial genetic diversity among ASFV isolates and their large genome size, infection susceptibility may differ substantially across macrophage polarization states (M0, M1, and M2), highlighting strain-specific tropism variation.

### ASFV affects the antigen-presenting function of DCs

DCs are potent antigen-presenting cells [112]. They can recognize, take up, and process antigens, followed by migration to regional lymph nodes to present processed antigens to lymphocytes, ultimately activating CD4^+^ or CD8^+^ T-cell responses [113]. However, the literature presents conflicting conclusions regarding the ability of ASFV to infect DCs. In a study by Zheng et al., pigs were infected with the genotype II ASFV CN/GS/2018 isolate, and single-cell sequencing was used to assess the viral load across different cell populations in the spleen. Notably, although no infected DCs were detected in the spleen from 3 to 7 days post-infection, the transcriptomes of the DCs showed significant changes in gene expression [106]. In another study, three ASFV strains with distinct virulence phenotypes (the virulent 22,653/14 strain, low-virulence NH/P68 strain, and avirulent BA71V strain) were shown to infect monocyte-derived DCs [114]. The ASFV late viral protein p72 was detected in DCs of the spleen, liver, and lungs in both domestic pigs and wild boars [115]. However, given the virulence variations among ASFV strains, further studies are required to confirm DC infectivity.

Notably, infection with the attenuated strain NH/P68 downregulates the expression of MHC-I and CD16 on infected moDCs, potentially impairing the effective initiation of the adaptive immune response. Simultaneously, NH/P68 infection induces the downregulation of CD80/86 costimulatory molecules on mature moDCs while promoting the upregulation of MHC class II molecules. Additionally, ASFV infection of immature and mature moDCs does not induce a strong cytokine response, and no statistically significant IFN-β, IL-1β, IL-6, IL-10, IL-12, IL-18, or TNF-α responses were observed between infected and uninfected moDC cultures [114].

Despite the central role that DCs play in shaping adaptive immunity against pathogens, few studies have analysed their interaction with ASFV. In our study (unpublished data), ASFV infection induced DC apoptosis in the peripheral blood of infected swine and markedly downregulated the surface expression levels of both MHC class I and class II molecules on DCs; therefore, the impact of ASFV on dendritic cells likely plays a more substantial role in the immune evasion strategies of the virus, and future investigations are needed to elucidate the underlying mechanisms involved.

### ASFV subverts B-cell function to evade humoral immunity

B cells express both MHC-I and MHC-II and are equipped with all the machinery required for antigen uptake, processing, and presentation, qualifying them as professional APCs [116]. B cells primarily mediate viral clearance through the antibody production, including both neutralizing and non-neutralizing antibodies. Neutralizing antibodies confer protection by directly interacting with viral surface proteins, blocking the infection of susceptible cells and curtailing viral dissemination.

Studies have reported marked lymphocytopenia in swine following ASFV infection, characterized by significant depletion of peripheral lymphocyte populations [117]. Infection with the genotype II ASFV CADC_HN09 strain induced both a significant reduction in B-cell counts and decreased CD21 MFI [104]. Given that CD21 serves as a marker for naive B-cell maturation and activation and facilitates complement activation, these findings suggest that ASFV infection may impair B-cell development and complement-dependent immune regulation.

Notably, the combination of B-cell depletion and CD21 downregulation on surviving B cells could collectively disrupt effective humoral immune responses, as CD21 critically regulates B-cell activation thresholds and antigen recognition [118]. Although ASFV does not directly infect B cells, indirect mechanisms such as cytokine dysregulation (e.g. IL-10 overproduction) and apoptotic signalling via Fas-FasL interactions may contribute to B-cell depletion [106, 119].

These perturbations lead to diminished neutralizing antibody titres against ASFV structural proteins such as p72 and p30, compromising viral clearance. According to the Immune Epitope Database (IEDB), several ASFV B-cell epitopes have been identified [120]. Despite the progress in ASFV antigenic epitope research supporting diagnostic and vaccine development, the epitope identification and application remain challenging [121]. A systematic approach to identify and validate B-cell epitopes with strong immunogenicity and cross-neutralizing activity is critical for rational vaccine design.

### γδ T cells in ASFV infection: virulence-related immune modulation

T lymphocytes express an antigen receptor (T-cell receptor, TCR) consisting of an αβ or γδ heterodimer. Like other ungulates, swine possess a high population of γδ T cells than many other species do: in young pigs, γδ T cells represent up to 50% of the total peripheral blood lymphocyte population, though their frequency decreases with age [122–124]. In pigs, γδ T cells exhibit characteristics of APCs, including expression of MHC class II molecules, which have been proposed as markers of porcine T cell activation [125, 126].

A recent study revealed that in domestic pigs infected with moderately virulent ASFV, elevated circulating γδ T-cell levels correlated with increased survival rates [127]. However, research by Xiong et al. showed that in ASFV-infected pigs displaying classical ASF symptoms, γδ T cells were severely depleted by the moderately virulent strain ASFV HB-2208 while a high proportion of γδ T cells persisted in asymptomatic pigs [105]. Notably, this protective immune response varies by virulence: during infections with highly virulent ASFV strains, γδ T cells not only decrease numerically but also lose their association with host survival [128]. This virulence-dependent variation suggests that ASFV strains may differentially affect γδ T-cell immunomodulatory functions, thereby impacting the host’s antiviral defence mechanisms. To date, little is known about the interaction of ASFV with porcine γδ T cells. Given their high frequency in pigs, γδ T cells likely influence ASFV infection outcomes, highlighting the need for further investigation.

## ASFV disrupts host antigen presentation to impair adaptive immunity

Viruses can exploit host gene expression mechanisms to modulate antigen presentation, with different pathogens employing distinct strategies to target antigen-presenting cells. Current research indicates that ASFV suppresses multiple stages of the antigen presentation; however, the precise mechanisms remain incompletely understood because of the complex genome and multifunctional virulence factors of this pathogen.

### ASFV disrupts MHC molecules to subvert adaptive immunity

Following the infection with genotype II ASFV CN/GS/2018, the splenic MHC-II gene expression (SLA-DQA1, SLA-DQB1, SLA-DRA, SLA-DRB1, SLA-DMA, SLA-DMB, and SLA-DOA) decrease in T/B cells, DCs, NK cells, monocytes, macrophages, and neutrophils at 5–7 days post-infection. Moreover, MHC-I expression was decreased in B cells, monocytes, macrophages, and DCs. Notably, decreased expression of MHC class II was observed in both permissive and nonpermissive cells throughout infection [106].

Although virulent ASFV infection increases the expression of MHC class I genes in macrophages, the bulk of the MHC class I molecules induced by ASFV infection remain intracellular [129]. Attenuated genotype I strains downregulate MHC class I expression in monocyte-derived macrophages and DCs, whereas virulent genotype I strains do not have this effect [114]. Infection with the ASFV-Georgia 2007 significantly reduced the expression of the cathepsin, impairing the antigen digestion and MHC-II loading, with the SLA-DMA and SLA-DMB downregulation by 12 hpi [17]. However, virulent ASFV Benin 97/1 infection caused no change in surface MHC-II expression on BMDM [130]. The above results indicate that ASFV may impair the early antigen presentation of APC through the modulation of SLA-I and -II, though strain-specific differences exist.

Interferon-mediated signalling not only induces the expression of host antiviral proteins but also stimulates antigen presentation through increased MHC expression [131]. As mentioned earlier, ASFV can inhibit type I and type II IFN signalling in a variety of ways to suppress antigen presentation. Both TGF-β and IL-10 are immunosuppressive cytokines that can downregulate the expression of MHC molecules [132]. O’Donnell et al. examined TGF-β levels on days 0, 7, and 14 after immunization of pigs with attenuated ASFV-G-9GL/UK. A few of the studied animals presented elevated TGF-β levels after immunization [133]. P P Powell et al. reported that the levels of TGF-β mRNA and protein increased post-ASFV infection [134]. IL-10 serum levels increased in domestic pigs infected with genotype II highly virulent isolates (SY18, HLJ/18) [135, 136]. High levels of IL-10 were detected in all pigs after challenge with the virulent genotype I ASFV Benin 97/1, and all pigs presented increased IL-10 concentrations before euthanasia [119]. The IL-10 levels in 11 wild boars inoculated with the genotype II virulent isolate Armenia07 prechallenge were significantly lower than those postchallenge [137]. Recently, early serum IL-10 surge after infection with the moderately virulent isolate Estonia2014 in domestic pigs, which presented more severe clinical signs and higher levels of several proinflammatory cytokines than SPF pigs did [137]. Therefore, IL-10 levels negatively correlate with survival. As TGF-β and IL-10 suppress innate and adaptive responses, high levels of these cytokines might favour viral pathogenesis by causing immune failure [138,139].

#### ASFV downgrades MHC-I antigen presenting pathway

Cytoplasmic proteins, including viral proteins, are processed through the class I antigen-presentation pathway. Many viruses evade adaptive immune recognition by targeting transporters associated with antigen presentation (TAPs) [140]. For example, the herpes simplex virus (HSV) ICP47 protein binds to TAPs and prevents the transfer of viral peptides into the endoplasmic reticulum (ER), thereby blocking their loading onto MHC-I [141–143]. Human cytomegalovirus (HCMV) US6 impairs TAP function by interfering with ATP binding to the transporter [144]. Furthermore, HCMV US2 and US11 can downregulate MHC-I expression by enhancing the degradation of the ER-localized MHC classI heavy α-chain [140].

Herpesviruses (HPV) have evolved yet another MHC class I immune evasion strategy: epitope evasion through selective depletion of high-affinity peptides that fit into the MHC-I binding cleft [145]. Computational enrichment analyses identified proteins depleted across the 30 most common human HLA-I genes, revealing that protein products of VZV ORFs 4, 9, 32, 61, 62, and 63 are significantly depleted in high-affinity peptides across many alleles, regardless of the VZV strain used [145]. Interestingly, four of these proteins – ORFs 4, 61, 62, and 63—are transcriptional regulators that are important in the early stages of infection and reactivation [146–150].

In ASFV infection, studies have demonstrated that the highly virulent strain Armenia2008 causes markedly decrease in maturation and reduces the surface transport of SLA-I in infected monocytes, despite stable SLA-I mRNA/protein levels and preserved antigen processing machinery. This impairment is accompanied by the loss of functional rough ER structures and prominent ER-associated aggregate formation. These cellular stresses ultimately lead to global protein translation arrest, mitochondrial dysfunction, and caspase-3-mediated apoptosis [151].

**Figure 2.**
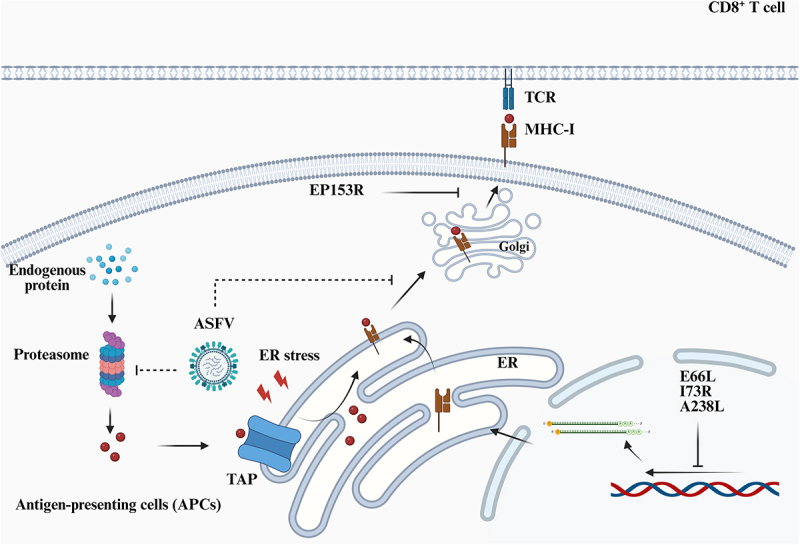
ASFV-mediated modulation of MHC class I antigen presentation. ASFV infection induces endoplasmic reticulum stress, leading to impaired MHC-I transport to the cell surface. The viral protein EP153R disrupts MHC-I transport from the Golgi to the plasma membrane, while E66L, I73R, and A238L suppress MHC-II expression via inhibition of the host translation machinery. The dashed lines denote putative ASFV-mediated inhibitory mechanisms involved in MHC-I downregulation that require experimental validation.

Notably, ASFV EP153R inhibits MHC-I membrane expression by impairing the exocytosis process while preserving MHC antigen synthesis or glycosylation [152]. Four ASFV-derived peptides from the p72 and P54 proteins were shown to bind swine leukocyte antigen 1 × 0101 (SLA-1 × 0101) [153]. Another study identified 21 highly conserved CD8^+^ epitopes in the pp62 protein, four of which demonstrated potential immunogenicity [154]. Although P72, P54 and PP62 are all late-stage ASFV proteins, their delayed expression may represent an immune evasion mechanism that postpones adaptive immunity. Figure 2 illustrates ASFV-encoded strategies for MHC-I suppression and potential immune evasion pathways.

#### ASFV downgrades MHC-II antigen presenting pathway

Endosomal and lysosomal viral proteins ingested by APCs are processed via the MHC class II antigen-presentation pathway, while viral proteins found in the cytoplasm and exocytic compartments can also be efficiently presented by MHC class II proteins [155, 156]. The MHC class II transactivator (CIITA), the master regulator of MHC class II expression, is regulated by IFN-γ signalling through the JAK/STAT pathway [156]. Kaposi’s sarcoma herpesvirus (KSHV)-encoded latency-associated nuclear antigen (LANA) downregulates CIITA transcription by suppressing the transcriptional activity of the CIITA promoters PIII and PIV [157]. Additionally, LANA binds RFX proteins, preventing the association of CIITA with MHC II promoters and further reducing MHC-II expression [158]. HCMV induces proteasomal degradation of JAK1, leading to MHC-II downregulation [159]. Similarly, HSV-1 targets STAT1 and STAT2 [156], and VZV targets STAT1α and JAK2, blocking downstream transcription of IRF1 and CIITA, thereby reducing MHC-II expression [160]. Moreover, VZV inhibits IFN-γ-induced MHC-II cell surface expression, potentially providing transient protection from CD4^+^ T-cell immune surveillance [160].

HSV also interferes with MHC-II expression post-translationally at different stages of MHC-II assembly and surface trafficking. For example, the KSHV replication and transcription activator (RTA) protein, an important regulator of the viral life cycle, can downregulate MHC II expression directly through the redirection of HLA-DRα to proteasomal degradation, and indirectly by increasing the expression of MARCH8, which results in the downregulation of its substrate HLA-DRα [161]. Furthermore, three HCMV-encoded proteins, US2, US3, and pp65, downregulate MHC-II expression during lytic infection and act at different stages of assembly and trafficking. US2 induces rapid proteasomal degradation of MHC-II heavy chains. US3 binds MHC-II αβ complexes, leading to mislocalization, and pp65 can traffic MHC-II complexes into perinuclear lysosomes for HLA-DRα degradation [156,162,163].

The transcription level of CIITA is decreased in ASFV-infected monocytes/macrophages [106,164]. ASFV might encode viral proteins or noncoding RNAs that interfere with the synthesis of CIITA or block its binding to the SLA-II promoter. Notably, MHC class II expression is dramatically decreased in both infected and bystander monocytes, suggesting cytokine-mediated regulation rather than direct viral infection effects [106].

Multiple ASFV proteins inhibit host translation. ASFV pE66L has been reported to have the ability to inhibit host translation. The transmembrane (TM) domain (amino acids 13 to 34) of pE66L is required for the inhibition of host gene expression, and the TM domain might help proteins localize to the ER to suppress translation via the PKR/eIF2α pathway [165]. ASFV pA238L inhibits the activation of the host nuclear transcription factors NF-κB and NFAT to regulate host gene expression [166], and pDP71L promotes the dephosphorylation of eukaryotic translation initiation factor 2 alpha (eIF2α) to prevent the protein synthesis [167]. ASFV pI73R is a Zα domain-containing nucleic acid-binding protein that localizes to the nucleus, where it broadly inhibits the host protein synthesis by blocking the nuclear export of cellular messenger RNA (mRNAs) [168].

ASFV proteins impair both IFN production and IFN-mediated antiviral responses, which indirectly affects the SLA-II generation. ASFV pI7L interacts with STAT1 and inhibits its Y98-dependent phosphorylation and homodimerization, thereby preventing the nuclear translocation of STAT1, resulted in decreased production of IFN-γ-stimulated genes [47]. The viral protein pH240R interacted with IFNAR1/2 to disrupt the complexes of IFNAR1-TYK2 and IFNAR2-JAK1. Additionally, pH240R inhibites the IFN-α-induced phosphorylation of IFNAR1, TYK2, and JAK1, ultimately suppressing the nuclear import of STAT1 and STAT2 [56].

SLA-II-mediated presentation of exogenous antigenic peptides requires proteasome activity. Yu et al characterized the interaction network of ASFV pD1133L in porcine kidney PK-15 cells and identified 1,471 host proteins potentially interacting with D1133L. KEGG pathway analysis revealed significant enrichment of proteasome pathway among these D1133L-host protein interactions [169]. Infected monocytes and macrophages showed a downregulation of the proteasome-associated proteins PSMA, PSMB, PSMC, PSMD, and PSME, suggesting possible impairment of proteasome function [106]. However, conflicting studies demonstrate that PSMA3 and PSMC1 interact with pMGF360-9L in vitro and their overexpression promotes ASFV replication, whereas MGF360-9L maintains the transcriptional levels of PSMA3 and PSMC1 [170]. Furthermore, Lucía’s study revealed that the ubiquitin – proteasome system is required for ASFV replication [171]. Therefore, further experiments are needed to clarify the precise impact of ASFV on proteasome activity.

**Figure 3.**
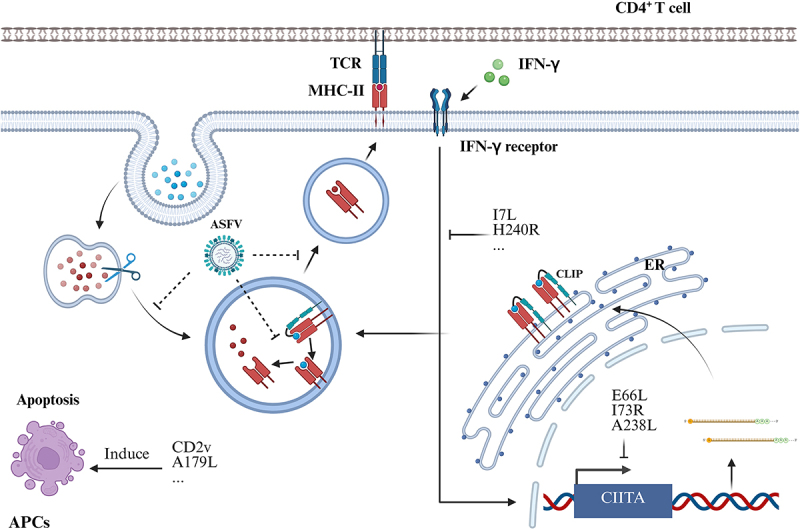
ASFV-mediated modulation of MHC class II antigen presentation. Viral proteins E66L, I73R, and A238L suppress host protein synthesis, thereby inhibiting MHC-II expression; while I7L and H240R impair MHC-II presentation through blockade of IFN-γ downstream signaling; and CD2v and A179L promote apoptosis, reducing MHC-II-positive macrophage populations. Dashed lines indicate potential ASFV-mediated inhibitory mechanisms contributing to MHC-II downregulation (pending experimentally validated).

A genome-wide CRISPR/Cas9 knockout screen in porcine cells identified RFXANK, RFXAP, SLA-DMA, SLA-DMB, and CIITA as essential genes for productive ASFV infection. These genes encode key components of SLA-II [172]. Notably, apoptosis in infected macrophages serves as a marker of acute ASF, as ASFV infection both leads to macrophage apoptosis and concurrently reduces SLA-II-positive cell numbers. As summarized in Figure 3, these findings delineate ASFV’s strategies on MHC-II-associated antigen presenting processes and potential immune evasion pathways.

### ASFV modulation of CD80/86 in immune evasion

ASFV infection downregulates CD80 transcript levels both in vivo and in vitro. However, transcriptional changes in CD86 following infection exhibit strain-specific differences. Infection with the Genotype II ASFV CN/GS/2018 strain downregulates CD86 expression in splenic monocytes and macrophages, while infection with genotype II ASFV-HLJ/18 strain upregulates CD86 expression in PAMs [36, 106]. The infection-mediated decrease in CD80/86 expression on mature moDCs suggests that ASFV may impair naive T cell activation through reduced costimulatory molecule expression [114]. For comparison, HSV-1 ICP22 directly binds the CD80 promoter to suppress its expression [173], and HSV-1 glycoproteins gD, gK, and gL collectively inhibit CD80 promoter activity.

### Viral exploitation of nonclassical MHC molecules in immune evasion

Nonclassical MHC molecules are structurally homologous to classical MHC molecules (MHC class I and II) but differ in antigen presentation, immunoregulation, or biological functions. Many viruses downregulate classical MHC-Ia proteins to prevent the presentation of viral antigens to CD8^+^ T cells, a strategy that can trigger NK cell “missing self” recognition. To counteract this, viruses often modulate NK cell responses by interacting with activating/inhibitory NK receptors or their ligands, which frequently include nonclassical MHC-Ib stress-induced proteins. Viral homologs of nonclassical class I MHC molecules play crucial roles in this process [174]. Herpesvirus-encoded Qa-1 mimics inhibit NK cell cytotoxicity through CD94/NKG2A receptor engagement [175]. The rhesus CMV Rh67 protein enables MHC-E surface expression, thereby evading the killing by NK cells [176]. HCMV UL18 blocks the priming of MHC-E – and MHC-II – restricted CD8^+^ T cells [177].

Research on ASFV and nonclassical MHC molecules remains limited. Current evidence suggests that the nonclassical MHC-II protein SLA-DM is crucial for ASFV replication [172]. While ASFV infection upregulates the transcription of the nonclassical MHC-I molecule SLA-6 in splenic macrophages, it downregulates the classical MHC-I molecules SLA-1, 2 and 3^106^potentially inhibiting NK cell targeting of infected cells. Further research is needed to elucidate the precise mechanisms by which ASFV regulates nonclassical MHC-I.

### ASFV induces organelle damage

ASFV infection causes structural and functional damage to multiple cellular organelles, including ER dilatation with cisternal collapse, disruption of the trans-Golgi network, impaired lysosomal acidification accompanied by size reduction, and decreased mitochondrial numbers [50, 129, 178–180]. Specifically, ASFV protein B117L modulates ER stress responses, activating the ATF6 branch and disrupting Ca^2 +^ homoeostasis, while EP152R induces ER swelling, activates the PERK/eIF2α pathway, and globally suppresses host protein synthesis in vitro [181]. The viral CD2v protein directly binds the TGN-associated AP-1 complex, which regulates cellular trafficking. This interaction likely mediates Golgi reorganization and redirects vesicular trafficking to support viral replication, particle assembly, and egress [182]. Furthermore, CP204L disrupts lysosomal function by sequestering VPS39 from the HOPS complex, thereby preventing lysosome clustering [183]. Concurrently, p17 enhances mitophagy via SQSTM1-TOMM70 interaction [50], whereas pE199L triggers mitochondrial apoptosis [184].

Both MHC class I and II molecules undergo essential processing in the ER followed by Golgi-mediated trafficking-processes that are disrupted by ASFV-induced structural and functional damage to these organelles. The observed reduction in mitochondrial numbers results in ATP depletion, severely compromising energy-dependent immunological processes such as antigen degradation and peptide transport. Moreover, impaired lysosomal function further reduces the efficiency of exogenous antigen processing. These ASFV-induced organelle damage further impair the antigen presentation process, resulting in suppression and delayed activation of innate and adaptive immune responses, thereby impairing the activation of CD4^+^ and CD8^+^ T cells and delaying viral clearance. This synergistic immune dysregulation ultimately promotes sustained viral persistence and systemic dissemination.

## Implications for vaccine design

Effective prevention remains the most important tool to keep ASF out of ASF-free regions, though current surveillance measures have shown limited effectiveness [185]. Developing effective and safe vaccines has therefore become crucial for ASF control [186]. While inactivated, subunit, mRNA, DNA, and viral-vectored vaccine candidates have failed to provide reliable protection, LAVs show promise by replicating in the hosts and inducing both humoral and cellular immune responses. However, vaccinated pigs may experience side effects including fever, skin necrosis, and joint swelling. Moreover, virulence reversion and inter-genotypic recombination raise serious biosafety concerns [14]. The commercially available vaccine ASFV-G-ΔI177L shows genetic and phenotypic instabilities during horizontal or vertical transmission [187].

A promising LAV design strategy involves deleting one or more virulence genes that suppress immune responses. The engineered OURT88/3 strain, with deletions in six MGF360 genes, two MGF505 members, a CD2-like protein, and a C-type lectin, elicited robust immune responses post-vaccination. However, the regulatory T cells (Tregs) showed a sustained upward trajectory throughout the 130-day observation period [119]. Notably, infection with the virulent Armenia08 strain also elevated FoxP3^+^ Tregs in domestic pigs and wild boars, particularly in blood and spleen. Although Treg responses vary during the acute infection, their increase suggests that gene-deleted viral strains may induce immunosuppression despite triggering immune responses.

The role of cellular immunity in ASFV infection is self-evident [188, 189]. Therefore, vaccines must elicit robust cellular immune responses. Many viruses target MHC class I-mediated antigen presentation to evade CD8^+^ T-cell immunity, and merging evidence suggests that viral immune evasion extends to the inhibition of nonclassical MHC-restricted CD8^+^ T-cell priming. For example, HCMV encoded proteins, including UL128, UL130, UL146, and UL147, effectively block both MHC class II and MHC-E-restricted CD8^+^ T-cell responses [190].

Current development of ASFV LAVs primarily focus on the serial deletion of virulence genes to attenuate the virus while preserving immunogenicity [191]. Although ASFV-mediated impact on antigen presentation remain poorly understood, rationally designed deletion mutants incorporating the removal of genes impairing the antigen presentation machinery may yield vaccine candidates with improved efficacy, safety, and tolerability profiles.

The two predominant ASFV vaccine platforms include gene-deleted LAVs and multiantigen subunit vaccines. However, the development of effective ASF vaccines facing challenges due to the limited understanding of the structural and functional properties of ASFV-encoded proteins, combined with the inherent complexity of the virus. The magnitude and durability of immune responses are critical determinants of vaccine efficacy. Targeted delivery of antigens to APCs has demonstrated substantially increased immunogenicity. A recent study reported that a self-assembling nanoparticle-based ASFV candidate vaccine (NanoFVax) designed to target DCs elicited broad and long-lasting immune responses with a favourable safety profile [192].

Compared to conventional virus-centred approaches, host-directed strategies may provide advantages in improved safety, longer-lasting immunity, broader protective potential, and better evasion resistance. Thus, the modulation of host immune pathways represents a promising alternative framework for future ASF vaccine design.

## Conclusion

ASF poses a major threat to the global swine industry, undermining sustainable development and creating significant challenges for pig production and socioeconomic stability. However, progress in understanding the molecular and immunological mechanisms of ASFV – immune system interactions has been limited by the lack of reliable cellular and animal models, severely hindering ASF control efforts. Monocytes/macrophages serve as the primary target cells for ASFV. Current research have predominantly focused on viral pathogenesis and macrophages responses, it often overlooks the effects of ASFV on other immune cells, especially APCs. Additionally, most studies use in vitro models that cannot fully mimic the complex intercellular interactions occurring in vivo.

Although ASFV does not directly infect APCs, emerging evidence suggests that infection-modulated intercellular communication networks and cytokine milieus could impair APC antigen-presenting capacity, consequently disrupting of adaptive immune responses. Therefore, systematic investigations into ASFV-APC crosstalk are crucial for understanding viral pathogenesis and transmission, which could inform new treatment and prevention strategies.

This review comprehensively integrates current knowledge on the impact of ASFV on APCs, analysing its strategies for disrupting antigen presentation. Through comparative analysis of immune evasion strategies employed by other viruses (e.g. interference with MHC class I/II trafficking, proteasomal degradation of antigenic peptides, and modulation of costimulatory signals), we highlight conserved mechanisms and propose ASFV-specific immune evasion hypotheses, offering a framework for targeted vaccine and therapeutic development.

## Data Availability

Data sharing is not applicable to this article, as no datasets were generated or analysed during the current study.
